# Digital Surveillance for Monitoring Environmental Health Threats: A Case Study Capturing Public Opinion from Twitter about the 2019 Chennai Water Crisis

**DOI:** 10.3390/ijerph17145077

**Published:** 2020-07-14

**Authors:** Jiangmei Xiong, Yulin Hswen, John A. Naslund

**Affiliations:** 1 Department of Biostatistics, Vanderbilt University, Nashville, TN 37203, USA; jx2348@columbia.edu; 2 Computational Epidemiology Lab, Harvard Medical School, Boston, MA 02215, USA; yulin.hswen@ucsf.edu; 3 Innovation Program, Boston Children’s Hospital, Boston, MA 02215, USA; 4 Department of Epidemiology and Biostatistics, University of California, San Francisco, CA 94158, USA; 5 Bakar Computational Health Sciences Institute, University of California, San Francisco, CA 94158, USA; 6 Department of Global Health and Social Medicine, Harvard Medical School, Boston, MA 02115, USA

**Keywords:** digital surveillance, disasters, crisis, water, public opinion, social media, natural language processing

## Abstract

Globally, water scarcity has become a common challenge across many regions. Digital surveillance holds promise for monitoring environmental threats to population health due to severe drought. The 2019 Chennai water crisis in India resulted in severe disruptions to social order and daily life, with local residents suffering due to water shortages. This case study explored public opinion captured through the Twitter social media platform, and whether this information could help local governments with emergency response. Sentiment analysis and topic modeling were used to explore public opinion through Twitter during the 2019 Chennai water crisis. The latent Dirichlet allocation (LDA) method identified topics that were most frequently discussed. A naïve Tweet classification method was built, and Twitter posts (called tweets) were allocated to identified topics. Topics were ranked, and corresponding emotions were calculated. A cross-correlation was performed to examine the relationship between online posts about the water crisis and actual rainfall, determined by precipitation levels. During the Chennai water crisis, Twitter users posted content that appeared to show anxiety about the impact of the drought, and also expressed concerns about the government response. Twitter users also mentioned causes for the drought and potential sustainable solutions, which appeared to be mainly positive in tone. Discussion on Twitter can reflect popular public opinion related to emerging environmental health threats. Twitter posts appear viable for informing crisis management as real-time data can be collected and analyzed. Governments and public health officials should adjust their policies and public communication by leveraging online data sources, which could inform disaster prevention measures.

## 1. Introduction

Water is essential for human life and a key part of any society. The United Nations describes water as “serving a crucial link between society and the environment” [1]. Fresh water is essential to activities in socioeconomic development, such as agriculture, hydroelectricity, food processing, industry, and human health [2]. The demand for fresh water has been increasing due to population growth, industrialization, and rising standards of living in many countries [3]. As a result of increasing domestic and industrial use of freshwater, as well as more frequent variations in weather due to climate change, water scarcity has become a common occurrence in many regions globally [4]. Shortages of water are also characterized by variability over geographic areas and over time [3]. Over the years, global warming has impacted freshwater availability negatively through many ways, including altering precipitation patterns [5].

Crisis brought by water shortage is not only detrimental to everyday activities, as mentioned above, but also negatively impacts economic development. For example, during a water crisis in Brazil, the shortage hindered hospital and business operations, as well as school activities [6]. As a result, local agriculture and hydropower output also suffered, and therefore the prices of commodities increased and the ability to engage in commerce was hampered [6]. Drought in Syria has led to large migration and social disruption [7], while in Lilongwe, the capital of Malawi, residents frequently suffer from water shortages to the point that they are not able to carry out essential hygiene practices and face increased health risks [8].

Water scarcity is a growing problem that disproportionately impacts lower-income countries [9], yet governments are poorly prepared to respond to these concerns and often fail to effectively monitor how water shortages impact the population. In the face of emerging threats related to water crises, governments require new methods for monitoring and responding to increasing risks. Water supply management has typically followed past hydrological patterns assuming relatively constant resource levels; yet, the changing climate has created significant disruptions [5]. Without close monitoring of water supply and policies that allow immediate follow-up in the event of sudden fluctuations and declines in water levels, water shortage problems can easily fall out of control. For instance, in the 2014 Brazil water crisis, the government was not actively monitoring and updating the water supply system; as a result, they were not able to act to resolve the water shortage through early response [6]. There are also additional problems caused by inefficient strategies aimed at preventing water shortages. One example is the huge south-to-north water transfer program in China, which did not successfully alleviate drought in the intended areas due to improper management of the water that was delivered [10].

Governments are in need of effective approaches for monitoring water supply as well as the ability to obtain real-time information to inform early response to mitigate potential water crises. The recent emergence of digital surveillance techniques may offer a novel approach that is ideally suited for addressing this need. Digital surveillance is performed through collecting vast streams of data from the digital trace, which refers to the data that are generated from everyday use of digital devices, that people leave on a continuous basis following their interaction with modern day technologies, and then extracting information from this data [11]. Social media platforms represent one common form of data that constitute a large part of the digital trace for many individuals, as usage of social media is predominant in most countries with more than 3.8 billion social media users as of 2020, which represents more than half of the world population [12]. Due to their copious and accessible nature, social media data can be used to extract useful information before, during, and after a potential water crisis [13].

There have been cases where digital surveillance has been used in disaster detection and water management. These approaches build on an extensive body of prior computational studies, where sentiment analysis involves extracting text data from social media platforms in order to capture user opinions and attitudes about a wide variety of topics [14]. For instance, sentiment analysis approaches have been tailored to different opinion categories and classification tasks, and have been compared against conventional data sources, and used to explore emotions and other distinct features of language used in online communication [14,15,16,17]. Of particular relevance to the current study, many studies have used sentiment analysis methods to identify the response to natural disasters from Twitter data. In Australia, a text-mining detector was developed to detect emergency situations from Twitter posts and is now being used by the Australian government [18], and based on this, a sensitive earthquake detector was built [19]. WaterScope is an online platform that combines water resource data and social media data to provide projections on water resources such as underground water levels and water concerns [20]. There are also more studies using digital surveillance methods for crisis management. For example, during Hurricane Sandy in the United States, analysis of large amounts of Twitter data revealed differences in risk perception across different population groups [21], while a case study for flooding in Hunan, China suggested that microblog data from Weibo, which shares similar features to Twitter, could reflect the severity of the flood impact and identify areas most in need of rescue work [22]. Similarly, opinion mining using the SentiSAIL tool was feasible for detecting positive and negative sentiments from German Twitter users during the 2013 Central European flood, which could generate insights for first responders [23]. In another study, a widely used sentiment analysis tool called the Valence Aware Dictionary and Sentiment Reasoner (VADER) could detect the unique characteristics of Twitter users and their patterns of influence based on their posts during Hurricane Harvey in the United States [24]. These studies highlight the promise of using social media platforms such as Twitter, and computational sentiment analysis methods to derive public opinions and attitudes about natural disasters and other environmental health threats.

To illustrate the potential use of digital surveillance for monitoring water shortages and for managing water crises, the purpose of this paper was to present a case study exploring the use of digital surveillance techniques for monitoring the 2019 water crisis in Chennai, India. This water shortage crisis lasted for several weeks and strongly impacted Chennai, the capital city of the state of Tamil Nadu, India. The water reservoir levels were shrinking steadily over the past year before the crisis, and the trigger of the crisis was the delay of the monsoon season [25]. In this paper, we use the 2019 Chennai water crisis as a case example in order to consider the following exploratory research aims with the goal of determining the potential utility of digital surveillance methods during a water shortage crisis: first, to identify the most common topics in online discussions related to the Chennai water crisis, followed by sentiment analysis of these topics. Second, to examine how online discussions related to the Chennai water crisis changed over time and whether these discussions showed any correlation with real-world changes in precipitation patterns, captured as level of rainfall. We intentionally employed a methodology involving widely accepted sentiment analysis techniques in this study because our goal was to offer flexibility and an approach that can be modified and adapted to fit other contexts or scenarios extending beyond monitoring water crises or natural disasters.

## 2. Methods

### 2.1. Context

The Chennai water crisis started on 19 June 2019 [26] due to the delayed start of the monsoon season and dried up water reservoirs. Tap water stopped flowing and people relied on water tankers, yet supply was severely limited. After almost one month following “day zero”, some relief for the situation was provided with the arrival of trucks and train cars loaded with water [27]. During the water crisis, restaurants, hotels, and businesses were forced to shut down [28]. Families were required to spend a sizable portion of their income on water as prices soared, while families in poverty had no choice but to cut down on their daily water usage to as low as 30–40 L, which is far less than the benchmark water supply of 135 L [29,30]. As a result of these challenges with obtaining water and mounting frustrations among residents of Chennai, there was an increasing number of disputes between local people over water-sharing [28]. Many of these concerns were also expressed on social media. Public figures including famous actors, athletes, and bloggers were tweeting about this crisis and raising awareness of climate change [31,32,33,34], while tweets criticizing local government further added to the debate [35].

### 2.2. Data Collection and Preprocessing

#### 2.2.1. Twitter Data

For this initial exploratory study, we used data collected from Twitter. Twitter is a popular social network platform where people can interact and discuss trending topics with posts containing up to 280 character limits, and the messages posted on the Twitter platform are called tweets [36,37]. By Twitter default, tweets are open to the public unless set specifically by users [38]. Given these features, Twitter is very popular when it comes to opinion sharing. Tweets are relatively more accessible when compared with content posted on other social media platforms, where user postings are generally not available publicly [39]. Tweets spanning the Chennai water crisis from 2 July 2019 to 22 July 2019 were accessed and collected through the Twitter application programming interface (API). We selected this three-week time frame during the ongoing Chennai water crisis because of the availability of data from Twitter during this time, and because during the second week, the occurrence of local precipitation changes was observed. Therefore, we expected that this timeframe would make it possible to detect changes in online conversations about the water crisis in response to changing precipitation levels.

The hashtag symbol (#) is used in front of the keywords in a tweet and can be used to identify the topic of a tweet [40]. To collect tweets that are most relevant to the Chennai Water Crisis, the collection was only limited to the tweets that contained the words “Chennai” and “Water” and a term related to drought or scarcity. Tweets under the following hashtags were collected: #ChennaiWaterCrisis, #ChennaiWaterScarcity, #Chennai&#Water, #Chennai&#drought. These hashtags were chosen because they contain keywords related to the water crisis and were trending during the crisis.

The Google translation API was used to translate non-English tweet texts [41]. The Google translation API was able to send back translated English text from the Google translation upon request when raw text was sent over to the cloud server [41]. Google Translate uses Google neural machine translation, which looks up sentence patterns in a broad context to produce the most accurate translation, and then rearrange the translation to fit into the grammar of the target language [42].

#### 2.2.2. Precipitation Data

To further check the relationship between the tweets and the drought, precipitation was used as a comparison [43]. Precipitation was collected through World Weather Online, where historical rainfall is recorded every three hours [44]. Daily precipitation in Chennai, India was collected and Pearson correlation tests were performed between precipitation and daily tweet frequency containing terms about the Chennai water crisis.

### 2.3. Data Analysis

In order to identify what people were talking about on Twitter regarding the Chennai water crisis, and how they were feeling towards these issues, we used sentiment analysis and topic modeling. Sentiment analysis can calculate how positive or negative each Tweet is, whereas topic modeling can identify different major topics among all the Tweets and categorize them under these topics. We also used topic heat, which makes it possible to categorize each tweet to an identified topic so as to further look into each topic.

#### 2.3.1. Exploratory Data Analysis

We examined the number of tweets per day as well as average tweet sentiment to see how people were feeling overall towards the Chennai water crisis. Sentiment analysis is a computational method that can identify to what extent a piece of text is positive or negative, and in this study, it was carried out using VADER [45]. VADER is specifically targeted to sentiment analysis on social media and can help identify tweets across three categories: positive, neutral, and negative [45]. VADER has been widely used across a range of studies using sentiment analysis of content posted on Twitter, including for monitoring outdoor air pollution [46], and understanding disaster resilience [47]. To measure the extent of negative Twitter sentiment for each day, the ratio of negative tweets against all tweets was calculated separately.

#### 2.3.2. Topic Modeling

Topic modeling belongs to natural language processing, and it identifies patterns in a text [48]. We employed latent Dirichlet allocation (LDA) in this study, which is a commonly used topic modeling approach. By applying LDA to text data, it returns topics in the form of groups of words with weight. Text data were tokenized, lemmatized, and stemmed to “group” words that have the same stem. As a result, the topics returned are word stems. The LDA model was implemented using Gensim [49], a package that includes LDA.

Gensim returns topics in the format of clusters “weight*word” connected by “+” signs (see example below). The number of topics returned is not a pre-fixed parameter, and in this study was selected manually based on interpretability and performance. If the number of topics was too small, the model with the higher number of topics was going to return more interpretable topics; if the number of topics was too big, some of the topics were going to be too similar to be identified. In this study, the number of topics was set to 8. Many times, the words in a topic appear confusing. To aid the interpretation of the topic, words returned by the LDA model were manually selected to represent that topic, and the following rules were applied in this study: (1) terms that were common in more than two different topics were dismissed as redundant; (2) verbs that did not specify a topic were also excluded; (3) if there were synonyms mentioned in one topic, only the one with the highest weight (the number before each word) was kept; (4) words that could not be found in VADER word collections were excluded; and (5) the top three weighted words were selected, after applying all the previous four rules. The lowest number of words that a topic has left after applying these first four rules is three; therefore, to keep all topics consistent, the number of words that represents the topic was capped at three. 

For instance, below we illustrate one of the topics returned by the LDA model:

Words: 0.036*“conserv” + 0.031*“crisi” + 0.028*“need” + 0.023*“home” + 0.019*“govern” + 0.018*“citi” + 0.018*“save” + 0.017*“go” + 0.016*“savewat” + 0.016*“wast”

Note that only the root of each word is kept within the topics returned by the LDA model, so the complete words from the original text are: conserve, crisis, need, home, government, city, save, go, savewater (as a Twitter hashtag), and waste. According to rules 1, 2, and 4: (1) the words “crisis”, “home”, “city”, and “save” are seen in several other topics; (2) the words “need” and “go” are used as verbs, and do not contain much information; and (4) the word “savewater” is not found in VADER. Therefore, the words representing this topic would be: “conserve”, “government”, and “waste”. It can be understood that this topic would be about conservation, government, and waste. To examine this interpretation, tweets under the topic can be sampled. The next section addresses how individual tweets were categorized into the topics identified.

#### 2.3.3. Identify Topic Heat

One single tweet can discuss one single topic or several topics at once. To determine which of the topics the tweet is most relevant to, we used a method called topic heat. Topic heat is a quantitative measure that describes how much a topic is being discussed. Topic heat is calculated using a similarity score generated by WordNet [50,51]. WordNet can calculate similarity between two word senses, “commonly accepted meaning of a word” [52], from a scale of 0 to 1, but only between words of the same part of speech. Although sometimes WordNet can give similarity between noun and verb, it borrows a hypernym of the verb to calculate similarity [50], which could lead to inaccuracy in this case.

#### 2.3.4. Text Preprocessing

Topics are word stems, which are not always included in the database of WordNet. Therefore, topic words generated by LDA are manually transferred into nouns. Tweets were tokenized, and each word was processed similarly, but using WordNet. WordNet can find a set of synonyms of the specific word (including the word itself), ranking in similarity with the word given, and the first noun will be selected. After this, all words that are accounted for in the similarity calculation will be nouns.

#### 2.3.5. Similarity Calculation and Accumulation

**Figure 1:**
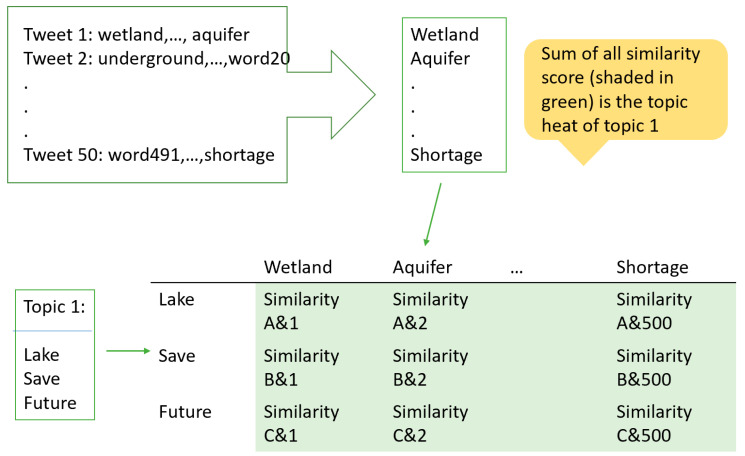
Calculation of topic heat for a given topic.

**Figure 2:**
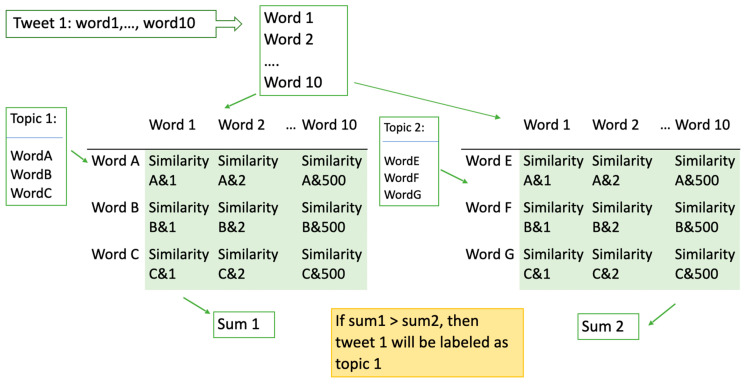
Process of categorizing tweets under topics using topic heat.

Topic heat is calculated in the form of total similarity of the words related to the topic. Consider a table, where column heads are the words in the topic and the row head are words from the targeted word corpus, as illustrated in Figure 1. The cell of the matrix is filled by the similarity between the row and column the cell is at. Then, do the same for all cells in the table, and the sum of all cells in the table will be the topic heat of the targeted word corpus. In this study, we calculated topic heats for all topics regarding each individual tweet (see Figure 2).

#### 2.3.6. Label Individual Tweets

To see which topic an individual tweet is mainly talking about, topic heat calculated in the last step is used. For every single tweet, its topic heat regarding all topics is available. The topic corresponding with the highest heat will be selected as the main topic of a given tweet. In other words, the tweet is labeled with that topic.

## 3. Results

### 3.1. Data Overview

Table 1 summarizes the number of tweets collected under each specified hashtag (group). Among a total of 5785 tweets about the Chennai water crisis, a total of 366 tweets (6.3%) were non-English and were translated. Table 2 shows the number of tweets and ratio of negative tweets in the three weeks over the studied period.

**Figure 3:**
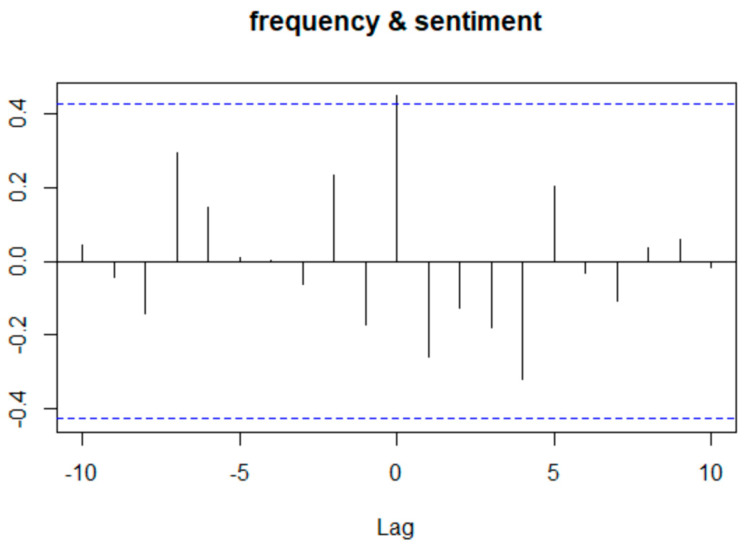
Auto correlation between tweet frequency and proportion of negative sentiment. Note: “Lag” on the horizontal axis refers to the number of time points that are different between A and B (in graph title). The black vertical line shows correlation between B at time x and A at time (x + lag), and the blue horizontal dash indicates the threshold of significance. If a black vertical line is taller than the blue horizontal dash, the correlation between A and B at that lag is considered statistically significant.

To explore the relationship between daily Twitter frequency and sentiment further, the daily negative sentiment fraction and number of tweets are lined up and cross-correlation was performed (Figure 3). Cross-correlation reveals the relationship between two time series at different time lag units. It can be seen that the statistically significant positive relationship (*p* = 0.040), where the correlation is higher than the significance threshold labeled by the blue line, is at zero lag. In other words, the fraction of negative sentiments appeared to increase as the number of discussions on Twitter increased simultaneously.

#### 3.1.1. Topic Modeling and Sentiment Analysis

In Table 3, all of the topics are listed, and the number of tweets is summarized under each topic. The number of tweets can be seen as an indication of how frequently a topic was discussed. Further, the average sentiment score, listed in the last column, shows how people on Twitter are talking about a given topic. We have included a sample text for each topic, as this can ensure that the tweet labeling is working correctly.

#### 3.1.2. Tweet Frequency and Precipitation

**Figure 4:**
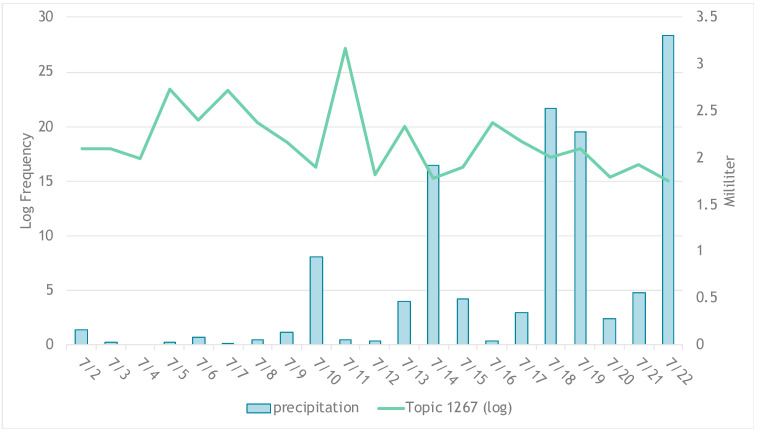
Precipitation and log frequency of tweets under topics 1, 2, 6, or 7 *. * Note: topics refer to the following: 1 (“*Lake save future*”), 2 (“*Conserve government waste*”), 6 (“*Pond rainwater nature*”), and 7 (“*Tap world important*”).

**Figure 5:**
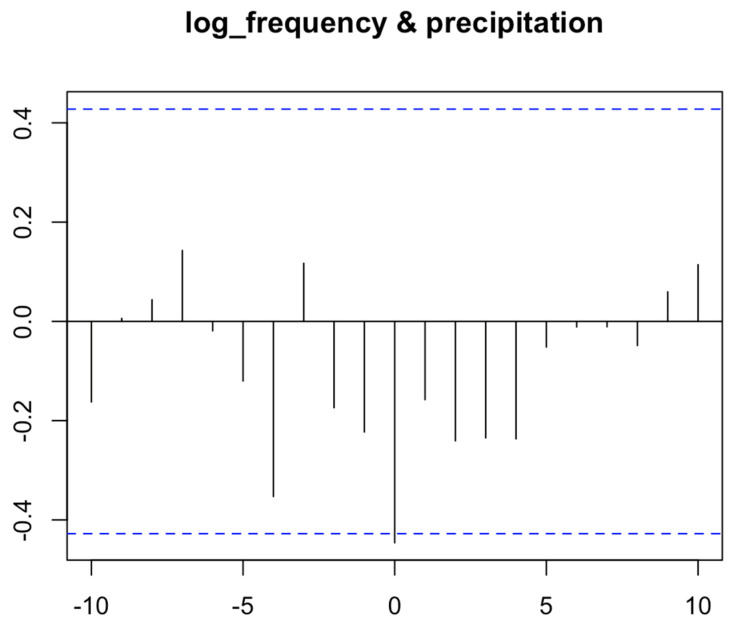
Cross correlation results from precipitation and log of the sum of tweets under topic 1, 2, 6, or 7. Note: This figure is interpreted in the same way as Figure 3, described above, where “Lag” on the horizontal axis refers to the number of time points that are different between A and B (log_frequency and precipitation, respectively). The black vertical line shows correlation between B at time x and A at time (x + lag), and the blue horizontal dash indicates the threshold of significance. If a black vertical line is taller than the blue horizontal dash, the correlation between A and B at that lag is considered statistically significant. The topics refer to the following: 1 (“*Lake save future*”), 2 (“*Conserve government waste*”), 6 (“*Pond rainwater nature*”), and 7 (“*Tap world important*”).

Although the correlation between the total number of tweets per day and precipitation is not significant (*p* = 0.119), it is observed that topic 1 (“*Lake save future*”), 2 (“*Conserve government waste*”), 6 (“*Pond rainwater nature*”), and 7 (“*Tap world important*”), which are with high numbers of tweets, appear to be negatively correlated with precipitation (Figure 4). Through the cross-correlation test (Figure 5), it is confirmed that the log frequency of the sum of the tweets under these topics has a significant negative correlation (*p* = 0.043) of −0.446 with precipitation simultaneously. In other words, tweets mentioning topics 1, 2, 6, or 7 decreased in number when precipitation levels increased during the Chennai water crisis.

## 4. Discussion

Digital surveillance has been previously used for monitoring water resources and natural disasters across diverse settings [19,20,21,22]. This study contributes to this growing body of evidence by exploring the case of the 2019 water crisis in Chennai, India using data captured from a widely used digital platform. Specifically, this study involved monitoring online discussions related to the Chennai water crisis on Twitter, a popular social media platform. The list of topics offers insights about what people appeared to be most concerned about in relation to the water crisis. Several of these topics include government management issues, daily water needs for individuals, environmental concerns such as climate change and late monsoon season, and importance of collecting rainwater and tap water and saving water for the future. Each of these topics was commonly mentioned during the three-week period that overlapped with the Chennai water crisis. Among these topics, government management issues were commonly represented by a large number of tweets, and were reflected by the highest proportion of negative tweets relative to the other topics. This could partly be explained by the nature of online conversations, and that political discussions are prevalent on Twitter, also in this case, the government was viewed as slow in responding to the crisis [53,54].

Generally, the tweets collected in this study were less negative when referring to water bodies or advocating for support and protection and water conservation. However, the tweets appeared to be more negative when talking about human behavior and the resulting impacts of the water crisis on daily life. From the sentiment analysis for different topics and samples from different tweets, people appeared to positively seek solutions to the water shortage problems, while generally not showing satisfaction with government action but feeling for the suffering of people during the crisis. These findings suggest that Twitter can be a potentially useful resource for local governments, as they could use the platform to collect authentic views from the public and draw from these insights to strengthen their communication campaigns, disaster response, and outreach efforts. This is consistent with prior studies showing that local governments can use social media content to inform decision-making. For example, tweets have been used after mass disasters for enabling quick damage assessment, and delivering messages through social media platforms and text messages, as was demonstrated in the 2010 Haiti earthquake for crisis mapping [55,56,57].

Exploring the relationship between the sentiment of the tweets collected in this study and actual precipitation levels is an important methodological strength, as it offers insights about how the number of discussions on Twitter fluctuates in response to changing rainfall levels, which is directly relevant to the source of the crisis. This corresponds to prior research showing that abnormal weather events can lead to increases in climate change-related conversations on Twitter [58]. The negative correlation observed between the log frequency of the most discussed and negative topics with precipitation levels suggests that the tweets examined in this study were mostly related to the Chennai water crisis. However, it should be pointed out that the correlation between the log frequency of all tweets and precipitation was not significant. This could indicate that either parts of the tweets collected are not entirely related to what we aimed to analyze, or that more indicators should be added alongside precipitation to generate a more precise estimate. Both speculations highlight the need for more in-depth work to advance computational methodologies using sentiment analysis and opinion mining of online data.

There have been studies using digital surveillance for emergency management when facing disasters, including from Australia, China, and the United States [19,21,22]. However, many existing studies have focused on the role of social media in emergency preparation, communication in emergencies, and detecting different user groups [59]. In particular, there have been fewer studies proposing mining public opinion from a popular social media platform like Twitter, specifically in terms of water management. There is one pilot study based on the 2014 California drought that explored Twitter data as well as Google trend data to obtain information for emergency management, in which they discovered that government policy during the drought influenced Twitter activity significantly, while prominent discussion could raise public awareness towards the drought [60]. However, additional follow-up studies have not looked into Twitter data in relation to water management [61,62].

Another study using Twitter examined the 2015 Chennai flooding [63], which has many similarities to our study towards advancing digital surveillance for disaster management in lower-resource settings such as in India. Importantly, this prior study highlighted that there was both noisy and relevant data on social media, and specifically identified the set of user posts that should be followed to obtain reliable and up-to-date messages regarding the crisis and areas most affected by the flooding [63]. This study combined with the findings reported here can contribute to advancing disaster response efforts in countries such as India.

The methods employed in this study can be executed swiftly, while the massive real-time information sharing on Twitter and accessibility to its data make timely reporting potentially more efficient and able to capture public perceptions [64]. This is especially important when considering methods for communicating with first responders, as it is critical to deliver messages as soon as possible to emergency responders and those working on the frontlines as the situation can change rapidly during an emergency. It may be difficult to employ such methods for this purpose using other social media platforms, with the exception of Weibo, which is similar to Twitter and is predominantly used in China. For example, another study demonstrated the use of YouTube comments to extract public opinion information on the Chennai water crisis and water crises more broadly in India [65]. Both this study and ours aimed at mining popular social media platforms for exploring public awareness about a severe water crisis. The difference lies in that the result of the study using YouTube focused on broader reception towards Indian water issues extending beyond the Chennai water crisis, while our results focused more specifically on the emergency response. In this case, information captured from the YouTube platform can be lagged, meaning that it can sit on YouTube for a considerable length of time before being viewed by the public. One advantage with Twitter is that tweets during an emergency can be collected rapidly, and this method can be applied across other emergency situations to obtain public opinions [13,66].

### Limitations

There are also limitations in this study that warrant consideration. For example, in terms of data, analysis results from tweets can only represent Twitter users, which limits the generalizability of these findings. This indicates that our results would exclude the segment of the population who does not have access to the internet and who do not use social media platforms like Twitter. This concern is particularly relevant in lower-resource settings, such as in India, where many individuals, especially those living in impoverished settings, do not have access to digital technologies or use social media. In terms of methodology, the key words needed for the tweet collection, number of topics returned by LDA, as well as topic key words were obtained through manual browsing and selection, which might not be applicable to all cases. Tweets are in different languages and possible inaccuracies in Google Translate may affect the outcome. Further, as we are expecting public opinion from local people who are impacted by the water crisis, there is concern that the locations of most tweets are not possible to be identified, as many users do not make their location information publicly available. This is an inherent limitation with data captured from Twitter. The topic model proposed is still naïve and issues need to be studied further, such as an alternative method that better quantifies word connections between verbs and nouns. This highlights an important area to advance natural language processing research, by ensuring better accuracy through detecting differences and similarities between nouns and verbs used in online posts. It is noteworthy that while there are limitations with using Twitter for digital surveillance, our findings add to an increasing number of studies showing that social media can effectively capture public opinions about natural disasters in lower-resource settings such as India. Social media platforms like Twitter can offer valuable insights that would be difficult to collect using traditional surveys or interview methods, and could be used to supplement existing public health and government response efforts.

## 5. Conclusions

This study sheds light on the topics that people are most concerned about and their emotions regarding these specific topics throughout a natural disaster in a low-resource setting. The method employed in this paper is flexible and can potentially be applied in different disaster settings for emergency response purposes, as well as emergency situations that extend beyond natural disasters to other public health threats. Future research can optimize the topic model proposed in this study, and try to incorporate tailored poststratification methods to alleviate the problem of underrepresentation. This is likely a particularly significant concern in the context of India, where many individuals may not have access to social media, and who are therefore not represented in the data captured from these popular social media platforms as part of digital surveillance efforts. Furthermore, our study highlights the need for research to expand on this approach and generate more in-depth and granular characterization of the impacts of a changing environment and crises on population health outcomes. When the accuracy of the method is improved, it can be adapted for a broader range of contexts and settings to enable prompt evaluation of public opinion and to inform disaster response. In the future, governments and local authorities could draw from the methods described in this case study to better understand public opinion and regions of greatest need as a reference to policy-making and communication with their people, especially in emergency situations where immediate information is desired.

## Figures and Tables

**Table 1:** Number of tweets collected under each key hashtag.

Hashtag	Number of Tweets Collected
#ChennaiWaterCrisis	4742
#ChennaiWaterScarcity	431
#Chennai #Water	524
#Chennai #drought	88
total	5785

**Table 2:** Number of tweets in each week and corresponding negative sentiment fraction.

Week	Number of Tweets	Negative Sentiment Fraction
July 2–July 8	1244	0.42
July 9–July 15	2097	0.29
July 16–July 22	2444	0.70

**Table 3:** Number of tweets, sample tweet, and sentiment of each topic.

Topic (Key Term)	Number of Tweets	Sample Text under Topic	Sentiment
1 Lake save future	1295	As cities expand, they’ve displaced wetlands and lakes that previously captured water and funneled it underground to recharge aquifers. Restoring and conserving urban water bodies could help cities in India prepare for future water shortages.	0.287
2 Conserve government waste	1625	When will we stop misusing our water bodies. Successive governments have been the biggest encroacher unfortunately.	0.969
3 Drink wash bath	87	We met Valarmathi, who has to live with her family on 5 buckets of water a day. That’s to bathe, cook, drink and wash clothes. It’s hard to imagine how you’d survive on that in any situation. But India is also in the middle of a heatwave	0.724
4 Crime climate action	536	This is a human problem exacerbated by climate change. It’s time for action	0.612
5 Monsoon poor reservoir	318	One of #India’s biggest cities has almost run out of #water. Very little rain, scorching temperatures, a late monsoon season, poor management and lax laws are being blamed.	0.406
6 Pond rainwater nature	1092	Just days after completing d restoration of the Pillayar Kovil Pond in Pallikarani, we were blessed with strong showers & today, the restored pond is holding water! When u give nature a chance to bounce back, Nature does it in Style!	0.306
7 Tap world important	832	An important thread Many of us take water for granted - but imagine living in a big city, where the taps have run dry. That’s the plight of people in one of India’s largest cities, #Chennai. And what’s going on there, could affect the wider world too.	0.061
